# COVIEdb: A Database for Potential Immune Epitopes of Coronaviruses

**DOI:** 10.3389/fphar.2020.572249

**Published:** 2020-09-02

**Authors:** Jingcheng Wu, Wenfan Chen, Jingjing Zhou, Wenyi Zhao, Yisheng Sun, Hanping Zhu, Pingping Yao, Shuqing Chen, Jianmin Jiang, Zhan Zhou

**Affiliations:** ^1^ College of Pharmaceutical Sciences, Institute of Drug Metabolism and Pharmaceutical Analysis and Zhejiang Provincial Key Laboratory of Anti-Cancer Drug Research, Zhejiang University, Hangzhou, China; ^2^ College of Computer Science and Technology, Zhejiang University, Hangzhou, China; ^3^ Key Lab of Vaccine, Prevention and Control of Infectious Disease of Zhejiang Province, Zhejiang Provincial Center for Disease Control and Prevention, Hangzhou, China; ^4^ Innovation Institute for Artificial Intelligence in Medicine, Zhejiang University, Hangzhou, China

**Keywords:** Coronavirus, Epitopes, Vaccine, Database, COVID-19

## Introduction

Two coronaviruses—severe acute respiratory syndrome coronavirus (SARS-CoV) and Middle East respiratory syndrome coronavirus (MERS-CoV)—have caused two large-scale pandemics in the past two decades (Drosten et al., 2003; Zaki et al., 2012). Now, the third coronavirus caused pandemic (COVID-19) is ongoing (Liu and Saif, 2020; Zhang N. et al., 2020). The 2019 novel coronavirus (2019-nCoV) which was first identified in Wuhan, China in December 2019, from patients with pneumonia is the very coronavirus (Zhu et al., 2020). Analysis of the viral genome has revealed that 2019-nCoV is phylogenetically close to SARS-CoV (Lu et al., 2020), as was named SARS-CoV-2. As of June 5, 2020, 6,640,960 people have been confirmed COVID-19, including 391,285 deaths (∼5.89% fatality rate) all over the world.

Because of the less cost-effective than treatment and reduce morbidity and mortality without long-lasting effects, vaccines are the most effective strategy for preventing infectious diseases (Zhang et al., 2019). However, there is still no approved vaccines for human coronaviruses (hCoV). There are several types of vaccines are under pre-clinical testing or clinical trials including inactivated vaccine, recombinant subunit vaccine, recombinant vector vaccine, and nucleic acid vaccine. In general, modern vaccines, such as recombinant subunit, peptide, and nucleic acid vaccines, are advantageous over other types of vaccines because of higher safety and less side effect, by inducing the immune system without introducing whole infectious viruses (Graham et al., 2013). Nucleic acid vaccines such as DNA vaccines and mRNA vaccines represent an innovative approach by direct injection of plasmids or mRNAs encoding the antigens, accompanied with a wide range of immune responses (Yang et al., 2004; Pardi et al., 2018). These advantages are applied with prophylactic vaccines and therapeutic vaccines to treat infectious diseases and cancers. For the development of modern vaccines, it is of critical importance to identify potential immune epitopes of 2019-nCoV, as well as other infectious pathogens.

Considering the seriousness of the recent outbreaks of zoonotic coronaviruses, therapeutic agents and vaccines for pan-coronaviruses should be developed to cope with the hCoV outbreaks in the present and in the future. Here, we predict all the potential B/T cell epitopes for SARS-CoV, 2019-nCoV, and MERS-CoV to provide potential targets for pan-coronaviruses vaccine development. The prediction are based on their proteins sequences. RaTG13-CoV is included because of its high homology with 2019-nCoV (96% whole genome identity (Zhou et al., 2020)). All the predicted results are stored in a database named COVIEdb (http://biopharm.zju.edu.cn/coviedb/).

## Methods

### Data Collection

The protein sequences of SARS-CoV, 2019-nCoV, MERS-CoV, and RaTG13-CoV are downloaded from NCBI (https://www.ncbi.nlm.nih.gov/).

The human leukocyte antigen (HLA) alleles used for T-cell epitopes prediction are derived from Zhou et al. which analyzed the HLA distribution of 20,635 individuals of Han Chinese ancestry (Zhou et al., 2016). The top 20 HLA I alleles of A, B, and C subtypes and HLA II alleles with frequency more than 5% are the final HLA datasets (**Supplementary Table 1**).

### B-Cell Epitope Prediction

The B-cell epitopes were predicted by the seven tools embedded in the Immune Epitope Database and Analysis Resource (IEDB) (Vita et al., 2015). More specifically, BepiPred-1.0 (Larsen et al., 2006), BepiPred-2.0 (Jespersen et al., 2017), Chou and Fasman beta turn prediction (Chou and Fasman, 1978), Emini surface accessibility scale (Emini et al., 1985), Karplus and Schulz flexibility scale (Karplus and Schulz, 1985), Kolaskar and Tongaonkar antigenicity scale (Kolaskar and Tongaonkar, 1990), and Parker hydrophilicity prediction (Parker et al., 1986) are used for predicting amino acid sites belonging to B-cell epitopes. The parameters are all set as default. The thresholds of each tool are listed in **Supplementary Table 2A**. In this study, only amino acids that be confirmed by at least five tools are considered as part of B-cell epitopes.

All tools give the score to define whether an amino acid is part of B-cell epitopes but not to define whether a peptide is B-cell epitopes. Here, we set B_score to quantify the possibility of a peptide to be B-cell epitopes, which is calculated as follows:

$$B\_score=\frac{\sum n_{a}}{L}$$

where *L* is the length of the peptide, *a* is the amino acid that belongs to the peptide, and *n_a_* is the number of tools convinced that amino acid is part of B-cell epitopes.

### T-Cell Epitope Prediction

The T-cell epitopes prediction were divided into two parts. One of them are presented by HLA I allele and would induce the activation of CD8+ T cells. This type of T-cell epitopes were predicted by NetMHCpan 4.0 (Jurtz et al., 2017), MHCflurry (Donnell et al., 2018), and DeepHLApan (Wu et al., 2019). Another type of T-cell epitopes presented by HLA II alleles were predicted by MixMHC2pred (Racle et al., 2019) and NetMHCIIpan (Karosiene et al., 2013), which would induce the activation of CD4+ T cells. The thresholds to define potential T-cell epitopes of each tool are listed in **Supplementary Table 2B**.

In the prediction of T-cell epitopes presented by HLA I alleles, all peptides with length range from 8 to 11 were selected and combined with previous HLA I alleles to create HLA-peptide pairs. It’s similar to predict that presented by HLA II alleles, with the difference that peptide length ranges from 15 to 28. Only HLA-peptide pairs satisfied with all thresholds of used tools would be convinced as potential T-cell epitopes in this study.

## Data Description

### Genome Organization of Four Coronaviruses

**Figure 1:**
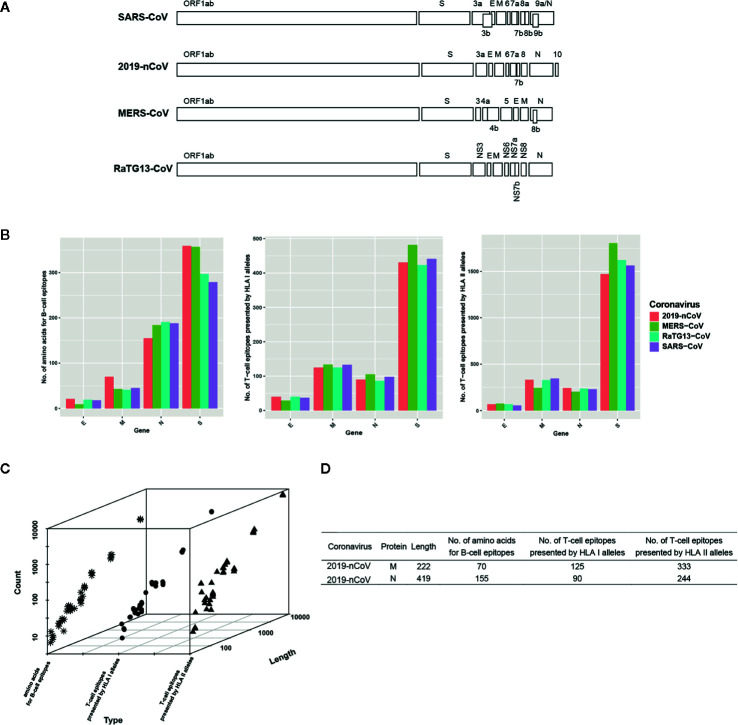
Genome organization of four coronaviruses and the characterization of predicted B/T-cell epitopes. **(A)** Genome organization of SARS-CoV, 2019-nCoV, MERS-CoV, and RaTG13-CoV. **(B)** The distribution of the predicted B/T epitopes of E, M, N, and S across four coronaviruses. **(C)** The relationship between protein length and the number of predicted B/T-cell epitopes. The snowflake indicates the number of predicted amino acids for B-cell epitopes, the circle indicates the number of predicted T-cell epitopes presented by HLA I alleles, and the triangle indicates the number of predicted T-cell epitopes presented by HLA II alleles. **(D)** The example proteins that have abnormal protein length and the number of B/T-cell epitopes relationship.

All selected coronaviruses have similar genome organization with coding genes of spike (S protein), envelope (E protein), membrane (M protein), nucleoprotein (N protein), and several open reading frames. SARS-CoV, 2019-nCoV, MERS-CoV, and RaTG13-CoV express 9, 8, 10, and 9 non-redundant protein coding genes, respectively (**Figure 1A**). In SARS-CoV, *orf3b* is overlapped with *orf3a* and E gene, *orf7b* is overlapped with *orf7a*, *orf8b* is overlapped with *orf8a*, and *orf9b* is part of *orf9a* (N gene). In 2019-nCoV, only *orf7b* is overlapped with *orf7a* and other genes are separated. In MERS-CoV, the *orf4b* is overlapped with *orf4a* and *orf8b* is part of N gene. In RaTG13-CoV, *ns7b* and *ns7a* are overlapped.

### Characterization of Predicted B/T-Cell Epitopes

Though some genes are overlapped, we predicted the potential B/T-cell epitopes of all genes because overlapped genes encode different proteins. Results show that the number of the predicted epitopes is different but similar among the homologous proteins of four coronaviruses (**Figure 1B** and **Supplementary Table 3**). Take the S protein as example, average 444 peptides are predicted as epitopes presented by HLA I alleles among four coronaviruses. The most is the S protein in MERS-CoV which occupies 482, the least is that in RaTG13 which occupies 423. Average 1,615 peptides are predicted as epitopes presented by HLA II alleles. The most is the S in MERS-CoV which occupies 1,804, the least is that in 2019-nCoV which occupies 1471. Average 323 amino acids are predicted as part of B-cell epitopes. The most is the S protein in 2019-nCoV which occupies 359, the least is that in SARS-CoV which occupies 279. The difference of predicted B/T-cell epitopes is minor in S. In other homologous genes, similar phenomenon occurs.

Normally, the number of predicted B/T-cell epitopes is positive correlated with the length of the proteins which genes translate (**Figure 1C**). However, there are also some exceptions that longer gene with less number of predicted B/T-cell epitopes, such as the M protein compared with the N protein in 2019-nCoV (**Figure 1D**). With nearly half length of encoded protein, M protein possesses more T-cell epitopes presented by both HLA I alleles and HLA II alleles than N protein, which indicates that M protein is preferred to be recognized by T cells than N protein. Besides, all proteins have predicted epitopes presented by HLA II alleles except ORF8a in SARS-CoV, which might be ascribed to its short length and less immunogenicity.

For better visualization of the predicted B/T cell epitopes, we create a database named COVIEdb (http://biopharm.zju.edu.cn/coviedb/). With four main pages “B-epitope”, “T-epitope”, “Peptide”, and “Validated”, researchers could find useful information easily and quickly. The predicted results of B-cell epitopes could be searched in “B-epitope” page. With the virus and gene selected, the corresponding predicted B-cell epitopes would appear. The predicted results of T-cell epitopes could be searched in “T-epitope” page. Similar with that in “B-epitope” page, coronavirus and protein are necessary. Besides, the type of T-cell epitopes should also be selected. Only the peptide-HLA pairs which satisfied thresholds of all tools would be showed in this page. The searchable data in the “Peptide” page is the combined result of previous predicted B-cell epitopes and T-cell epitopes. In this page, the only selectable parameter is the protein. The “Validated” page containing the predicted B/T epitopes that have been validated by recently literatures (Le Bert et al., 2020; Zhang B. Z. et al., 2020). To date, there are only 116 validated epitopes in the “Validated” page. However, with the growing research on coronaviruses, more validated data would be added to the “Validated” page.

### Shared B/T-Cell Epitopes

Though the evolution rate of human coronavirus is fast, we try to find out B/T-cell epitopes conserved and shared in different coronaviruses for the pan-coronavirus vaccine development. Based on the predicted B-cell epitopes and T-cell epitopes, we found 77 peptides that exist in all coronaviruses have the potential to induce T-cell activation and 10 of them with B_score larger than 4 (**Table 1** and **Supplementary Table 4**). In particular, the peptide YFKYWDQTY from ORF1ab could be presented by 7.33% people, which might be a good candidate for vaccine design.

**Table 1:** The potential T-cell epitopes with B_score larger than 4.

Protein	Peptide	HLA able to present peptide	Peptide location indifferent coronaviruses	B_score	HLA I frequency	HLA II frequency
ORF1ab	KPGGTSSGDATTAYA	DQA1_05_05:DQB1_03_01	SARS-CoV:5045_5059 2019-nCoV:5068_5082 MERS-CoV:5054_5068 RaTG13-CoV:5067_5081	4.98	0	0.42
ORF1ab	TSSGDATTAY	HLA-B15:01	SARS-CoV:5049_5058 2019-nCoV:5072_5081 MERS-CoV:5058_5067 RaTG13-CoV:5071_5080	4.98	1.68	0
ORF1ab	SSGDATTAY	HLA-B15:01 HLA-B35:01 HLA-B15:02	SARS-CoV:5050_5058 2019-nCoV:5073_5081 MERS-Cov:5059_5067 RaTG13CoV:5072_5080	4.97	3.4	0
ORF1ab	KPGGTSSGDATTAYAN	DQA1_05_05:DQB1_03_01	SARS-CoV:5045_5060 2019-nCoV:5068_5083 MERS-CoV:5054_5069 RaTG13-CoV:5067_5082	4.73	0	0.42
ORF1ab	GGTSSGDATTAYAN	DQA1_05_05:DQB1_03_01	SARS-CoV:5047_5060 2019-nCoV:5070_5083 MERS-CoV:5056_5069 RaTG13-CoV:5069_5082	4.7	0	0.42
ORF1ab	GGTSSGDATTAYANS	DQA1_05_05:DQB1_03_01	SARS-CoV:5047_5061 2019-nCoV:5070_5084 MERS-CoV:5056_5070 RaTG13-CoV:5069_5083	4.47	0	0.42
ORF1ab	GTSSGDATTAYANS	DQA1_05_05:DQB1_03_01	SARS-CoV:5048_5061 2019-nCoV:5071_5084 MERS-CoV:5057_5070 RaTG13-CoV:5070_5083	4.36	0	0.42
ORF1ab	GGTSSGDATTAYANSV	DQA1_05_05:DQB1_03_01	SARS-CoV:5047_5062 2019-nCoV:5070_5085 MERS-CoV:5056_5071 RaTG13-CoV:5069_5084	4.19	0	0.42
ORF1ab	YFKYWDQTY	HLA-B15:01 HLA-B15:02 HLA-C07:02	SARS-CoV:4655_4663 2019-nCoV:4678_4686 MERS-Cov:4664_4672 RaTG13CoV:4677_4685	4.08	7.33	0
ORF1ab	MGWDYPKCDR	HLA-A31:01	SARS-CoV:4984_4993 2019-nCoV:5007_5016 MERS-Cov:4993_5002 RaTG13CoV:5006_5015	4.07	1.21	0

All the T-cell epitopes shared in four coronaviruses are located in ORF1ab. However, the S protein of the coronavirus is the most important protein where the receptor binding domain (RBD) located. So, we further investigated the shared epitopes that located in S protein. There are 265 potential epitopes in S protein shared by three coronaviruses and 35 of them with B_score larger than 5 (**Supplementary Table 5**). The peptides VYDPLQPEL and TVYDPLQPEL even have B_score larger than 6. To note, though these two peptides differs only one amino acid, the HLA alleles that can bind with them are different. VYDPLQPEL can be presented by HLA-C07:02, HLA-C04:01, and HLA-C14:02, with overall 8.26% frequency in Chinese Han population, while TVYDPLQPEL can be presented by HLA-A02:06 and HLA-C12:03, with 2.44% frequency. The two peptides are different in the aspect of epitopes, but we could take them as one when choosing the vaccine target, which indicates the feasibility of the peptides to be potential pan-coronavirus vaccine target.

We believe that these results and the developed database could benefit not only the vaccine (especially the multiple-epitope vaccine which could protect from various coronavirus) development but also provide the targets for drug design such as neutralizing antibody on 2019-nCoV and the possible coronavirus outbreak in the future.

## Data Availability Statement

The datasets presented in this study can be found in online repositories. The names of the repository/repositories and accession number(s) can be found below: COVIEdb (http://biopharm.zju.edu.cn/coviedb/).

## Author Contributions

ZZ and JJ conceived of the idea and supervised the study. JW, WC, and JZ performed the epitope prediction. JW constructed and maintained the database and web interface. WZ, YS, HZ, PY, and SC participated in the data analysis. JW and ZZ wrote the manuscript. All authors contributed to the article and approved the submitted version.

## Funding

This work was supported by the Key R&D Program of Zhejiang Province (Grant No. 2020C03010), the National Natural Science Foundation of China (Grant No. 31971371), the Zhejiang Provincial Natural Science Foundation of China (Grant No. LY19H300003), and the Fundamental Research Funds for the Central Universities of China.

## Conflict of Interest

The authors declare that the research was conducted in the absence of any commercial or financial relationships that could be construed as a potential conflict of interest.

## References

[B1] Chou P. Y. Fasman G. D. (1978). “Prediction of the secondary structure of proteins from their amino acid sequence,” in Advances in Enzymology and Related Areas of Molecular Biology. Ed. Meiste A. (New Jersey: John Wiley & Sons, In), 45–148. 10.1002/9780470122921.ch2 364941

[B2] Donnell T. J. O. Rubinsteyn A. Bonsack M. Riemer A. B. Laserson U. Hammerbacher J. (2018). MHCflurry : Open-Source Class I MHC Binding Tool. Cell Syst. 7, 129–132.e4. 10.1016/j.cels.2018.05.014 29960884

[B3] Drosten C. Günther S. Preiser W. Van der Werf S. Brodt H. R. Becker S. (2003). Identification of a novel coronavirus in patients with severe acute respiratory syndrome. N. Engl. J. Med. 348, 1967–1976. 10.1056/NEJMoa030747 12690091

[B4] Emini E. A. Hughes J. V. Perlow D. S. Boger J. (1985). Induction of hepatitis A virus-neutralizing antibody by a virus-specific synthetic peptide. J. Virol. 55, 836–839. 10.1128/jvi.55.3.836-839.1985 2991600 PMC255070

[B5] Graham R. L. Donaldson E. F. Baric R. S. (2013). A decade after SARS: Strategies for controlling emerging coronaviruses. Nat. Rev. Microbiol. 11, 836–848. 10.1038/nrmicro3143 24217413 PMC5147543

[B6] Jespersen M. C. Peters B. Nielsen M. Marcatili P. (2017). BepiPred-2.0: Improving sequence-based B-cell epitope prediction using conformational epitopes. Nucleic Acids Res. 45, W24–W29. 10.1093/nar/gkx346 28472356 PMC5570230

[B7] Jurtz V. Paul S. Andreatta M. Marcatili P. Peters B. Nielsen M. (2017). NetMHCpan-4.0: Improved Peptide–MHC Class I Interaction Predictions Integrating Eluted Ligand and Peptide Binding Affinity Data. J. Immunol. 199, 3360–3368. 10.4049/jimmunol.1700893 28978689 PMC5679736

[B8] Karosiene E. Rasmussen M. Blicher T. Lund O. Buus S. Nielsen M. (2013). NetMHCIIpan-3.0, a common pan-specific MHC class II prediction method including all three human MHC class II isotypes, HLA-DR, HLA-DP and HLA-DQ. Immunogenetics 65, 711–724. 10.1007/s00251-013-0720-y 23900783 PMC3809066

[B9] Karplus P. A. Schulz G. E. (1985). Prediction of chain flexibility in proteins - A tool for the selection of peptide antigens. Naturwissenschaften 72, 212–213. 10.1007/BF01195768

[B10] Kolaskar A. S. Tongaonkar P. C. (1990). A semi-empirical method for prediction of antigenic determinants on protein antigens. FEBS Lett. 276, 172–174. 10.1016/0014-5793(90)80535-Q 1702393

[B11] Larsen J. E. P. Lund O. Nielsen M. (2006). Improved method for predicting linear B-cell epitopes. Immunome Res. 2:2. 10.1186/1745-7580-2-2 16635264 PMC1479323

[B12] Le Bert N. Tan A. T. Kunasegaran K. Tham C. Y. L. Hafezi M. Chia A. (2020). SARS-CoV-2-specific T cell immunity in cases of COVID-19 and SARS, and uninfected controls. Natures 584, 457–462. 10.1038/s41586-020-2550-z 32668444

[B13] Liu S. L. Saif L. (2020). Emerging Viruses without Borders: The Wuhan Coronavirus. Viruses 12, E130. 10.3390/v12020130 31979013 PMC7077218

[B14] Lu R. Zhao X. Li J. Niu P. Yang B. Wu H. (2020). Genomic characterisation and epidemiology of 2019 novel coronavirus: implications for virus origins and receptor binding. Lancet 395, 565–574. 10.1016/S0140-6736(20)30251-8 32007145 PMC7159086

[B15] Pardi N. Hogan M. J. Porter F. W. Weissman D. (2018). mRNA vaccines-a new era in vaccinology. Nat. Rev. Drug Discov. 17, 261–279. 10.1038/nrd.2017.243 29326426 PMC5906799

[B16] Parker J. M. R. Guo D. Hodges R. S. (1986). New Hydrophilicity Scale Derived from High-Performance Liquid Chromatography Peptide Retention Data: Correlation of Predicted Surface Residues with Antigenicity and X-ray-Derived Accessible Sites. Biochemistry 25, 5425–5432. 10.1021/bi00367a013 2430611

[B17] Racle J. Michaux J. Rockinger G. A. Arnaud M. Bobisse S. Chong C. (2019). Robust prediction of HLA class II epitopes by deep motif deconvolution of immunopeptidomes. Nat. Biotechnol. 37, 1283–1286. 10.1038/s41587-019-0289-6 31611696

[B18] Vita R. Overton J. A. Greenbaum J. A. Ponomarenko J. Clark J. D. Cantrell J. R. (2015). The immune epitope database (IEDB) 3.0. Nucleic Acids Res. 43, D405–D412. 10.1093/nar/gku938 25300482 PMC4384014

[B19] Wu J. Chen W. Zhou J. Zhao W. Chen S. Zhou Z. (2020). COVIEdb : A database for potential immune epitopes of coronaviruses. bioRxiv. [Preprint] 10.1101/2020.05.24.096164 PMC7898897 33628169

[B20] Wu J. Wang W. Zhang J. Zhou B. Zhao W. Su Z. (2019). DeepHLApan: A deep learning approach for neoantigen prediction considering both HLA-peptide binding and immunogenicity. Front. Immunol. 10, 2559. 10.3389/fimmu.2019.02559 31736974 PMC6838785

[B21] Yang Z. Y. Kong W. P. Huang Y. Roberts A. Murphy B. R. Subbarao K. (2004). A DNA vaccine induces SARS coronavirus neutralization and protective immunity in mice. Nature 428, 561–564. 10.1038/nature02463 15024391 PMC7095382

[B22] Zaki A. M. Van Boheemen S. Bestebroer T. M. Osterhaus A. D. M. E. Fouchier R. A. M. (2012). Isolation of a novel coronavirus from a man with pneumonia in Saudi Arabia. N. Engl. J. Med. 367, 1814–1820. 10.1056/NEJMoa1211721 23075143

[B23] Zhang C. Maruggi G. Shan H. Li J. (2019). Advances in mRNA vaccines for infectious diseases. Front. Immunol. 10, 594. 10.3389/fimmu.2019.00594 30972078 PMC6446947

[B24] Zhang B. Z. Hu Y. F. Chen L. L. Yau T. Tong Y. Hu J. (2020). Mining of epitopes on spike protein of SARS-CoV-2 from COVID-19 patients. Cell Res. 30, 702–704. 10.1038/s41422-020-0366-x 32612199 PMC7327194

[B25] Zhang N. Wang L. Deng X. Liang R. Su M. He C. (2020). Recent advances in the detection of respiratory virus infection in humans. J. Med. Virol. 92, 408–417. 10.1002/jmv.25674 31944312 PMC7166954

[B26] Zhou F. Cao H. Zuo X. Zhang T. Zhang X. Liu X. (2016). Deep sequencing of the MHC region in the Chinese population contributes to studies of complex disease. Nat. Genet. 48, 740–746. 10.1038/ng.3576 27213287

[B27] Zhou P. Yang X. L. Wang X. G. Hu B. Zhang L. Zhang W. (2020). A pneumonia outbreak associated with a new coronavirus of probable bat origin. Nature 579, 270–273. 10.1038/s41586-020-2012-7 32015507 PMC7095418

[B28] Zhu N. Zhang D. Wang W. Li X. Yang B. Song J. (2020). A novel coronavirus from patients with pneumonia in China 2019. N. Engl. J. Med. 382, 727–733. 10.1056/NEJMoa2001017 31978945 PMC7092803

