# Corrigendum: COVIEdb: A Database for Potential Immune Epitopes of Coronaviruses

**DOI:** 10.3389/fphar.2020.646111

**Published:** 2021-02-08

**Authors:** Jingcheng Wu, Wenfan Chen, Jingjing Zhou, Wenyi Zhao, Yisheng Sun, Hanping Zhu, Pingping Yao, Shuqing Chen, Jianmin Jiang, Zhan Zhou

**Affiliations:** ^1^ Institute of Drug Metabolism and Pharmaceutical Analysis and Zhejiang Provincial Key Laboratory of Anti-Cancer Drug Research, College of Pharmaceutical Sciences, Zhejiang University, Hangzhou, China; ^2^ College of Computer Science and Technology, Zhejiang University, Hangzhou, China; ^3^ Key Lab of Vaccine, Prevention and Control of Infectious Disease of Zhejiang Province, Zhejiang Provincial Center for Disease Control and Prevention, Hangzhou, China; ^4^ Innovation Institute for Artificial Intelligence in Medicine, Zhejiang University, Hangzhou, China

**Keywords:** coronavirus, epitopes, Vaccine, database, COVID-19

In the original article, there was an incorrect reference.

A correction has been made to the reference for MixMHC2pred in the **Methods** section. It was incorrectly written as Chen, B., Khodadoust, M. S., Olsson, N., Wagar, L. E., Fast, E., Liu, C. L., et al. (2019). Predicting HLA class II antigen presentation through integrated deep learning. Nat. Biotechnol. 37, 1332–1343. doi: 10.1038/s41587-019-0280-2. It should be Racle, J., Michaux, J., Rockinger, G. A., Arnaud, M., Bobisse, S., Chong, C., et al. (2019). Robust prediction of HLA class II epitopes by deep motif deconvolution of immunopeptidomes. Nat. Biotechnol. 37, 1283–1286. doi: 10.1038/s41587-019-0289-6.

The authors apologize for this error and state that this does not change the scientific conclusions of the article in any way. The original article has been updated.

